# Suppression of Rice Cryptochrome 1b Decreases Both Melatonin and Expression of Brassinosteroid Biosynthetic Genes Resulting in Salt Tolerance

**DOI:** 10.3390/molecules26041075

**Published:** 2021-02-18

**Authors:** Ok Jin Hwang, Kyoungwhan Back

**Affiliations:** Bioenergy Research Center, Department of Biotechnology, College of Agriculture and Life Sciences, Chonnam National University, Gwangju 61186, Korea; smilax@jnu.ac.kr

**Keywords:** cadmium, blue-light photoreceptors, brassinosteroids, CRY1b, melatonin, transgenic rice

## Abstract

We investigated the relationship between the blue-light photoreceptor cryptochrome (CRY) and melatonin biosynthesis by generating RNA interference (RNAi) transgenic rice plants that suppress the *cryptochrome 1b* gene (*CRY1b*). The resulting *CRY1b* RNAi rice lines expressed less *CRY1b* mRNA, but not *CRY1a* or *CRY2* mRNA, suggesting that the suppression is specific to *CRY1b*. The growth of *CRY1b* RNAi rice seedlings was enhanced under blue light compared to wild-type growth, providing phenotypic evidence for impaired CRY function. When these *CRY1b* RNAi rice plants were challenged with cadmium to induce melatonin, wild-type plants produced 100 ng/g fresh weight (FW) melatonin, whereas *CRY1b* RNAi lines produced 60 ng/g FW melatonin on average, indicating that melatonin biosynthesis requires the CRY photoreceptor. Due to possible feedback regulation, the expression of melatonin biosynthesis genes such as *T5H*, *SNAT1*, *SNAT2*, and *COMT* was elevated in the *CRY1b* RNAi lines compared to the wild-type plants. In addition, laminar angles decreased in the *CRY1b* RNAi lines via the suppression of brassinosteroid (BR) biosynthesis genes such as *DWARF*. The main cause of the BR decrease in the *CRY1b* RNAi lines seems to be the suppression of CRY rather than decreased melatonin because the melatonin decrease suppressed *DWARF4* rather than *DWARF*.

## 1. Introduction

Light-dependent melatonin synthesis occurs in some plant species, whereas in other plants, melatonin exhibits a nocturnal increase similar to that in animals [1,2,3,4]. Melatonin is broadly implicated in many physiological activities in plants, where it mostly serves as a potent free radical scavenger, signal molecule, or hormone via the mitogen-activated protein kinase (MAPK) signaling pathway [5,6,7,8,9]. Although a plant melatonin receptor was recently reported [10], its integrity remains controversial and requires further study [11]. The major known functions of melatonin are plant defense responses against both biotic and abiotic stresses [12,13], plant growth, and reproduction [7,14]. Briefly, melatonin is closely involved in conferring tolerance against endoplasmic reticulum stress [15,16], salt [17,18], pathogens [19,20], high temperatures [21,22], high light stress [23], and other stresses [24]. Melatonin also plays roles in plant growth and reproduction, including the synthesis of secondary metabolites [25,26], germination [27], seed longevity [28], hormone synthesis [29,30], fruit yield [31], and other processes [32].

The melatonin biosynthesis pathways have been well-characterized with the successful cloning of relevant biosynthesis genes [8]. It is clear that melatonin induction is dependent on light based on experiments in which rice plants were challenged with cadmium, which is the best inducer of melatonin [2]. The pivotal gene responsible for melatonin induction under light is that encoding tryptophan decarboxylase (TDC), the first committed step enzyme in melatonin biosynthesis, which converts tryptophan into tryptamine. The optimal light for the maximum induction of melatonin was found to be a combination of red and blue light, rather than red or blue alone; far-red light is unable to induce melatonin in response to cadmium treatment in rice, indicating that melatonin synthesis is predominantly associated with photosynthesis rather than with other photoreceptors such as phytochromes [33].

**Figure 1:**
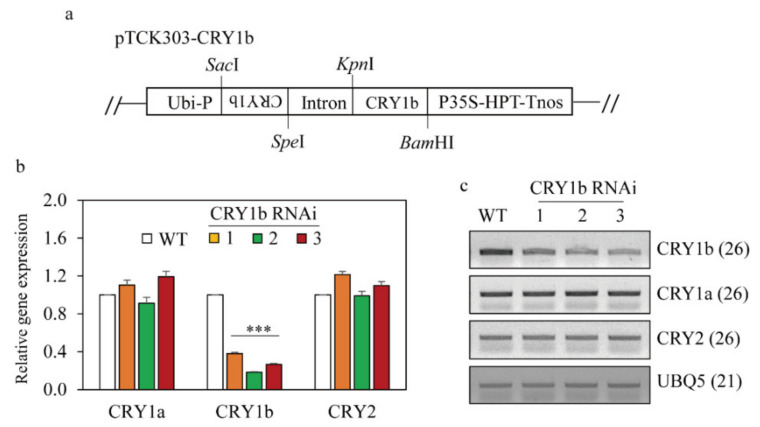
The structure of binary vector pTCK303 and the generation of *CRY1b*-suppressed transgenic rice plants. (**a**) Schematic diagram of the pTCK303:CRY1b binary vector. (**b**) Results of quantitative real-time polymerase chain reaction (PCR) analyses. (**c**) Results of reverse transcription-PCR analyses of the wild-type and transgenic (T2) lines. Rice seeds were germinated in half-strength Murashige and Skoog (MS) medium and grown for 8 days at 28 °C under a 14-h light/10-h dark cycle. Total RNA was extracted from these seedlings. *CRY1b*, *Oryza sativa cryptochrome 1b*; *Ubi-P*, *maize ubiquitin promoter*; *HPT*, gene encoding *hygromycin phosphotransferase*. Asterisks (***) indicate significant differences from the wild type (*p* < 0.001). GenBank accession numbers are AB024337 (*CRY1a*), AB098568 (*CRY1b*), AK065669 (*CRY2*), and Os03g13170 (*UBQ5*). Numbers in parentheses indicate the numbers of PCR cycles.

Light is indispensable for plant growth via photosynthesis, during which mainly red (720 nm) and blue (450 nm) wavelengths are absorbed via photosynthetic pigments, including chlorophyll [34]. Light is also an important signal that influences plant life and death from germination to reproduction. Plants have evolved specific photoreceptors that respond to different light wavelengths. For instance, phytochromes absorb red (600–700 nm) and far-red (700–750 nm) light as part of their biological functions, whereas phototropins and cryptochromes (CRY) sense ultraviolet A/blue light (390–500 nm). The first molecular genetic analysis of the possible involvement of photoreceptors in melatonin synthesis was recently reported, in which rice phytochrome mutants exhibited decreased melatonin synthesis compared to wild-type plants, confirming a previous report that melatonin synthesis requires light [33]. However, the possible involvement of CRY in melatonin biosynthesis in plants remains unclear [35]. Here, we generated transgenic rice plants that suppressed rice *CRY1b* to investigate if CRY are also involved in melatonin synthesis in rice. The rice genome contains a small *CRY* gene family that includes *CRY1a* (AB024337), *CRY1b* (AB098568), and *CRY2* (AK065669). CRY1a exhibits greater homology with CRY1b (75% identity), whereas CRY1a and CRY1b exhibit less homology with CRY2, with amino-acid sequence identities of 46% and 47%, respectively [36]. Of the three *CRY* genes, *CRY1b* was reported to be associated with blue light-dependent phenotypes in *Arabidopsis* and rice [37]. Therefore, we chose rice *CRY1b* to develop transgenic RNAi rice plants to examine whether CRY is involved in melatonin biosynthesis (Figure 1a). Due to the high homology between CRY1a and CRY1b, we selected a C-terminal portion of *CRY1b* that exhibits greater sequence diversity from that in *CRY1a* for RNA silencing.

## 2. Results and Discussion

### 2.1. Generation of Transgenic Rice Plants Suppressing Rice CRY1b

**Figure 2:**
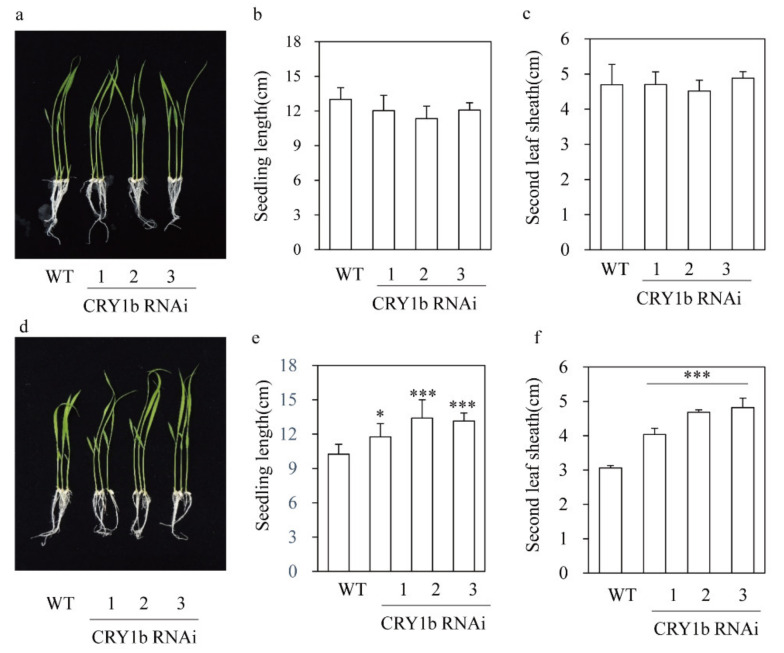
Seedling phenotypes of wild-type (WT) and *CRY1b* RNAi transgenic rice. (**a**) Representative seedling phenotypes, (**b**) shoot lengths, and (**c**) second leaf sheath lengths of plants grown under cool fluorescent light. (**d**) Representative seedling phenotypes, (**e**) shoot lengths, and (**f**) second leaf sheath lengths of plants grown under blue light. Rice seeds were germinated in half-strength MS medium and grown for 8 days at 28 °C under a 14-h light/10-h dark cycle. Asterisks denote significant differences (* *p* < 0.05, *** *p* < 0.001) as determined by Tukey’s HSD post-hoc tests. Lines 1–3 are RNAi rice plants with downregulated *CRY1b* genes.

From four independent T1 lines, we selected three homozygous T2 transgenic lines (Figure 1b,c). *CRY1b* was significantly suppressed in the three *CRY1b* RNAi lines, whereas *CRY1a* and *CRY*2 mRNA levels were comparable to those in the wild type according to qRT-PCR and semiquantitative RT-PCR analyses. These data indicated the successful, specific suppression of *CRY1b* using RNAi. The lengths and second leaf sheath lengths of 8-day-old wild-type and *CRY1b* RNAi seedlings grown in half-strength Murashige and Skoog (MS) medium under a 12-h light/12-h dark regime with cool fluorescent light were similar (Figure 2a–c). However, seedling length was longer in the *CRY1*b RNAi lines than in the wild type when seedlings were grown under blue light, as was the length of the second leaf sheath (Figure 2d–f). Notably, wild-type seedling length decreased under blue light versus cool fluorescent light because blue light inhibits seedling growth in monocots, including rice [37,38]. Consistent with this, our transgenic *CRY1b* RNAi seedlings exhibited significantly reduced inhibition of seedling growth and second leaf sheath length, confirming that our *CRY1b* RNAi rice plants had a functional defect in the blue-light photoreceptor (CRY). In contrast to results obtained for *CRY1*b RNAi, the overexpression of *CRY1b* induced an increased responsiveness to blue light, resulting in severe seedling growth inhibition compared to the wild type [36,37].

### 2.2. Melatonin Levels in CRY1b RNAi Rice Seedlings

**Figure 3:**
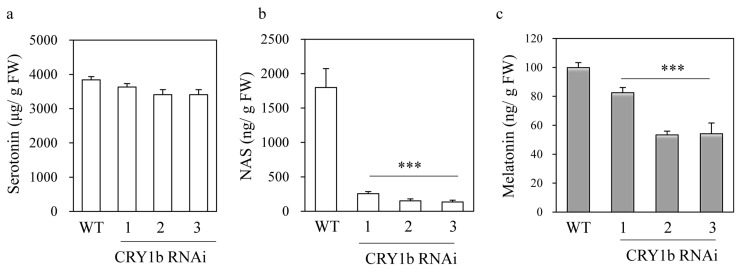
Serotonin, *N*-acetylserotonin, and melatonin contents in WT and *CRY1b* RNAi lines. (**a**) Serotonin, (**b**) *N*-acetylserotonin (NAS), and (**c**) melatonin levels in response to cadmium treatment under cool fluorescent light. Eight-day-old rice seedlings were challenged rhizosperically with 0.5 mM CdCl_2_ for 3 days under cool fluorescent light at a rate of 60 μmol m^−2^ s^−1^. Values are presented as the means ± standard deviations (SDs) of three independent experiments. FW, fresh weight. *** *p* < 0.001, Tukey’s HSD post-hoc test.

**Figure 4:**
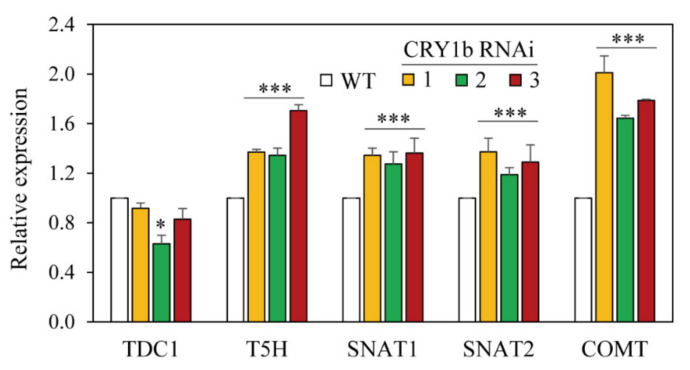
Quantitative real-time PCR analyses of melatonin biosynthesis genes. *TDC1*, tryptophan decarboxylase 1; *T5H*, tryptamine 5-hydroxylase; *SNAT1*, serotonin *N*-acetyltransferase 1; *SNAT2*, serotonin *N*-acetyltransferase 2; *COMT*, caffeic acid *O*-methyltransferase. * *p* < 0.05 and *** *p* < 0.001 vs. the WT. GenBank accession numbers are AK069031 (*TDC1*), AK071599 (*T5H*), AK059369 (*SNAT1*), AK068156 (*SNAT2*), AK064768 (*COMT*), and Os03g13170 (*UBQ5*).

To determine whether the blue-light photoreceptor (CRY) was associated with melatonin biosynthesis, 8-day-old rice seedlings were challenged with cadmium to induce melatonin. As shown in Figure 3, melatonin levels decreased two-fold in *CRY1b* RNAi rice lines 2 and 3 and by 20% in line 1 compared to those in the wild type (Figure 3c). A drastic decrease in *N*-acetylserotonin (NAS) levels was observed in all *CRY1b* RNAi lines, with NAS contents 10-fold less in RNAi lines (166 ng/g fresh weight (FW)) than in the wild type (1750 ng/g FW) (Figure 3b). Conversely, serotonin levels in the *CRY1b* RNAi lines were indistinguishable from those in the wild type (Figure 3a). These data indicate that the blue-light photoreceptor is involved in melatonin biosynthesis when rice seedlings are challenged with cadmium. Next, we measured the expression of melatonin biosynthesis gene mRNA in 7-day-old rice seedlings in the absence of cadmium. TDC is the first enzyme to participate in melatonin biosynthesis, followed by tryptamine 5-hydroxylase (T5H), which synthesizes serotonin from tryptamine. Serotonin is then acetylated into NAS by serotonin *N*-acetyltransferase (SNAT), which has two isogenes in the rice genome—*SNAT1* and *SNAT2*. The last enzyme in melatonin synthesis is NAS *O*-methyltransferase (ASMT), which catalyzes the conversion of NAS into melatonin. The caffeic acid *O*-methyltransferase (COMT) exhibiting ASMT enzyme activity plays pivotal roles in melatonin synthesis in rice [39]. The expression of the melatonin biosynthesis genes *T5H*, *SNAT1*, *SNAT2*, and *COMT* was all elevated in the *CRY1b* RNAi lines, whereas that of *TDC1* was not (Figure 4). The high levels of melatonin biosynthesis gene expression in the *CRY1b* RNAi lines suggest that feedback regulation compensates for low melatonin levels.

### 2.3. Decreased Leaf Angle in the CRY1b RNAi Rice Seedlings

**Figure 5:**
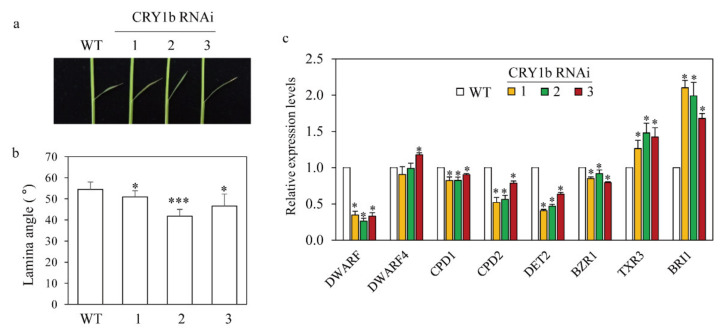
Lamina joint angles and transcript induction profiles of brassinosteroid (BR)-related genes in WT and *CRY1b* RNAi (1–3) rice plants. (**a**) Photograph of representative lamina joints in plants grown for 10 days in soil pots. (**b**) Lamina joint angles in WT and transgenic rice seedlings. (**c**) Quantitative real-time PCR analysis of the expression of various BR-related genes in WT and *CRY1b* RNAi plants. GenBank accession numbers are Os03g0602300 (*DWARF*), Os03g12660 (*DWARF4*), Os11g0143200 (*CPD1*), Os12g0139300 (*CPD2*), Os01g0851600 (*DET2*), Os07g39220 (*BZR1*), AP005859 (*TXR3*), AK101085 (*BRI1*), and Os03g13170 (*UBQ*5). Lines 1–3 are RNAi rice plants with downregulated *CRY1b* genes. * *p* < 0.05, *** *p* < 0.001, Tukey’s HSD post-hoc test.

Blue light promotes leaf bending by upregulating brassinosteroid (BR) biosynthesis genes in rice [40]. Similarly, the overexpression of *CRY* genes increases the leaf angle in rice, implying a positive relationship between CRY and leaf angle [36]. Additionally, melatonin is positively associated with the leaf angle in rice by regulating BR levels [29]. To investigate the role of *CRY1b* in determining leaf angle and BR-related gene expression, lamina angles of the second leaves of 10-day-old seedlings grown in soil pots were measured. As shown in Figure 5, the *CRY1b* RNAi lines had smaller leaf angles than the wild type. To examine the possible contribution of BR-related genes to the decreased leaf angles in the *CRY1b* RNAi lines, we quantified the expression of various BR-related genes. *DWARF*, *DWARF4*, *CPD* (*CONSTITUTIVE PHOTOMORPHOGENIC DWARF*), and *DET2* (*DEETIOLATED2*) are BR biosynthesis genes. DWARF converts 6-deoxo-catasterone into catasterone via a C6 oxidation reaction, whereas DWARF4 converts 6-oxo-campestanol into cathasterone via C22 hydroxylation. *CPD* and *DET2* encode C3 oxidase and 5-reductase, respectively [40]. BR-responsive genes include *BZR1* (*BRASSINAZOLE RESISTANT1*; a BR receptor), *TXR3* (*XYLOGLUCAN ENDOTRANSGLYCOSYLASE RELATED*), and *BRI1* (*BRASSINOSTEROID-INSENSITIVE 1*; a transcription factor for BR biosynthesis). *TXR3* is a BR-inducible gene encoding a cell wall-loosening enzyme. All BR biosynthesis genes except for *DWARF4* were downregulated in the *CRY1b* RNAi lines compared to the wild type, suggestive of reduced BR synthesis. Our data implicate that *CRY* suppression leads to BR suppression in rice. Intriguingly, the decrease in melatonin caused by the suppression of *SNAT2* led to a decrease in BR via the suppression of *DWARF4* [29]. Collectively, we attribute the BR decrease in the *CRY1b* RNAi lines mainly to the downregulation of CRY, rather than a decrease in melatonin caused by the suppression of *CRY1b*. The expression of *BZR1* also decreased in the *CRY1b* RNAi lines relative to the wild type, indicating that BR levels decreased in the *CRY1b* RNAi lines. Contrary to the results observed for BR biosynthesis genes, BR-responsive genes such as *TXR3* and *BRI1* were significantly upregulated in the *CRY1*b RNAi lines relative to the wild type, suggesting that these genes are regulated by feedback from reduced levels of endogenous BR.

### 2.4. CRY1b RNAi Rice Exhibits Salt Tolerance, Possibly via a Decrease in BR

**Figure 6:**
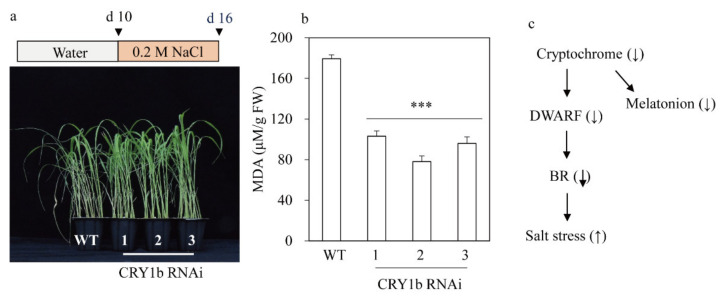
Effects of salt treatment on *CRY1b* RNAi rice plants. (**a**) Photograph of representative phenotypes after salt treatment. (**b**) Malondialdehyde (MDA) levels in WT and transgenic rice plants. (**c**) Proposed model of CRY-mediated salt tolerance. Values are presented as the means  ±  SDs of three independent experiments. FW, fresh weight; d, day. *** *p* < 0.001, Tukey’s HSD post-hoc test.

Exposure to blue light may increase BR synthesis, possibly via CRY in *Arabidopsis* and rice [40,41]. In fact, the overexpression of *CRY1a* conferred hypersensitivity against salt in *Arabidopsis*, possibly through ABA (abscisic acid) signaling [41]. BR levels have been shown to be negatively associated with abiotic stresses in plants [42,43,44]. Therefore, a decrease in endogenous BR levels or signaling can lead to abiotic stress tolerance in plants via the ABA and BR signaling pathways [29,41,44]. To test whether the *CRY1b* RNAi lines showing the reduced expression of BR-biosynthetic genes than the wild type confer abiotic tolerance or not, the *CRY1b* RNAi lines were challenged with salt, and their responses were monitored. As shown in Figure 6, *CRY1b* RNAi rice exhibited a salt-tolerant phenotype, reflected by reduced production of malondialdehyde (MDA) compared to the wild type. Our data strongly suggest that *CRY1b* RNAi rice produces less BR than the wild type, which accounts for the enhanced salt tolerance.

## 3. Materials and Methods

### 3.1. Plant Growth Conditions

Rice (*Oryza sativa* cv. Dongjin) seeds were sterilized and grown in soil or half-strength Murashige and Skoog (MS) under cool daylight fluorescent lamps (60 μmol m^−2^ s^−1^) (Philips, Amsterdam, Netherlands) in a 14-h light/10-h dark photoperiod or blue light (15 μmol m^−2^ s^−1^) (STECH LED, Gyeonggi-do, Korea) at 28 °C/24 °C (day/night). The angles of the lamina joint were measured in the second leaf of 10 d-old rice seedlings grown in soil. For tolerance assay in response to salt stress, 10-d old seedlings were exposed to 200 mM NaCl for 6 days.

### 3.2. Generation of CRY1b Suppression Transgenic Rice by RNA Interference (RNAi)

The pTCK303 binary vector was used to suppress rice *CRY1b* gene expression as previously described [45]. Briefly, a C-terminal 288 bp *CRY1b* cDNA fragment was amplified by PCR using the following primer set: *CRY1b-F* 5′-ACT AGT GAA AAT TTCCGTACCACT-3′ (*Spe*I site underlined) and *CRYIb-R* 5′-GAG CTC CTG GGA TAA TTG ACT CCA-3′ (*Sac*I site underlined), with the cDNA templates synthesized from the total RNA from rice seedlings. The *CRY1b* PCR product was first subcloned into the T&A cloning vector (T&A:CRY1b; RBC Bioscience, New Taipei City, Taiwan) for further cloning experiments. From the T&A:CRY1b plasmid, the antisense *CRY1b* insert was digested by *Sac*I and *Spe*I, whereas the sense *CRY1b* insert was digested by *Kpn*I and *Bam*HI in the T&A:CRY1b vector. The antisense *CRY1b* fragment was first ligated into the pTCK303 vector. Thereafter, the sense fragment of the *CRY1b* insert was sequentially ligated into the pTCK303 vector plasmid harboring the corresponding *CRY1b* antisense fragment (Figure 1a). The resulting pTCK303:CRY1b RNAi binary vector was transformed into *A*. *tumefaciens* LBA4404, and into rice (*Oryza sativa* cv. Dongjin), as previously described [46,47]. In brief, in order to generate transgenic rice plants, scutellum-derived calli from rice (*O. sativa* cv. Dongjin) were co-cultured with *Agrobacterium* harboring the pTCK303:CRY1b binary vector for 3 days, during which T (transfer)-DNA is incorporated into the rice genome. Thereafter, rice calli were screened on N6 selection medium containing 2 mg/L 2,4-D and 50 mg/L hygromycin for 30 days in the dark to generate transgenic calli, and transferred to regeneration media containing 3 mg/L BAP (6-benzylaminopurine), 1 mg/L NAA (1-naphthalene acetic acid), and 50 mg/L hygromycin for shoot and root development. The resulting rice seedlings were grown in a paddy field for obtaining T1 seeds.

### 3.3. Cadmium Treatment

Eight-day old seedlings were incubated in water containing 0.5 mM CdCl_2_ (Sigma-Aldrich, St. Louis, MO) under cool daylight fluorescent lamps (60 μmol m^–2^ s^–1^) (Philips, Amsterdam, The Netherlands) for 3 days at 28 °C. The leaves and stems were harvested for further analyses.

### 3.4. Quantitative Real-Time Polymerase Chain Reaction (qRT-PCR) Analysis

Total RNA of rice plants was isolated using a NucleoSpin RNA Plant Kit (Macherey-Nagel, Düren, Germany). First-strand cDNA was synthesized from 2 μg of total RNA using MG MMLV Reverse Transcriptase (MGmed, Inc., Seoul, Korea) and an oligo dT_18_ primer at 42 °C for 1 h. qRT-PCR was performed in a Mic qPCR Cycler system (Biomolecular Systems, Queensland, VIC, Australia) with specific primers and the Luna Universal qPCR Master Mix (New England Biolabs, Ipswich, MA, USA), as described previously. The expression of genes was analyzed using Mic’s RQ software (Biomolecular Systems) and normalized to *ACT1*. Semi-quantitative RT-PCR was performed as described previously [2].

### 3.5. Quantification of Serotonin, N-acetylserotonin, and Melatonin

Frozen samples (0.1 g) were pulverized to a powder in liquid nitrogen using the TissueLyser II (Qiagen, Tokyo, Japan). For serotonin and *N*-acetylserotonin, rice samples were extracted with 1 mL of methanol for 1 h at room temperature. The extracts were centrifuged for 10 min at 12,000× *g*, and the supernatants (20 µL) were subjected to high performance liquid chromatography (HPLC) using a fluorescence detector system (Waters, Milford, MA, USA), as described previously [2]. For melatonin measurement, the samples were extracted with 1 mL of chloroform for 16 h at 4 °C. The extracts were centrifuged for 10 min at 12,000× *g*, and the supernatants (20 µL) were completely evaporated and dissolved in 0.1 mL of 40% methanol, and 20-µL aliquots were subjected to HPLC using a fluorescence detector system (Waters, Milford, MA, USA), as described previously [2]. All measurements were performed in triplicate.

### 3.6. Malondialdehyde Measurement

The rice samples (50 mg) were ground and extracted with 1.5 mL of reaction buffer (containing 0.5% thiobarbituric acid (TBA) and 20% trichloroacetic acid (TCA)). The extracts were centrifuged for 15 min at 12,000× *g*, and the resulting supernatants were collected for measurement. The supernatant was boiled for 25 min at 95 °C, and placed for 5 min in ice. MDA content was determined at 440, 532, and 600 nm wavelengths using a spectrophotometer (OPIZEN POP-BIO). MDA was quantified using a molar extinction coefficient of 156/nM/cm.

### 3.7. List of Primers for qRT-PCR

The following primers were utilized for qRT-PCR and semiquantitative RT-PCR. *CRY1a* (forward 5′-AGA TGG AAG TTG ACC GAG-3′; reverse 5′-AGG CTG GAA GAG CAC CTG CG-3′), *CRY1b* (forward 5′-AGA TGG AAG TTG ACC GAG -3′; reverse 5′-AGG CTC AAA ATT CAC ACG GGT-3′), *CRY2* (forward 5′-ACG AGC TCT GTA GCA AAC TCA-3′; reverse 5′- TTT TCG TTG TCT TGA AAT GCT-3′), *TDC1* (forward 5′-GCG AGG GTG AAA CCT TCC A-3′; reverse 5′-GCG AGC CGG TGG AGT CC-3′), *T5H* (forward 5′-CCT CGT CCT GGA CAT GTT CGT C-3′; reverse 5′-ATG GCG AAC GTG TTG ATG AAC AC-3′), *SNAT1* (forward 5′-CAG TAG AGC CAC CAT CAG CA-3′; reverse 5′-ATC CCA CCT TGT CGC ATA AA-3′), *SNAT2* (forward 5′-GTC TGG GAC GTG GTC GTG-3′; reverse 5′-GTT GCC TTG AGC GGT AGA AG-3′), *COMT* (forward 5′-CCT GCT CGC CTC CTA CAA-3′; reverse 5′- ATG CCC TCG TTG AAG ACG-3′), *DWARF* (forward 5′-GGA GAA GAA CAT GGA ATC AC -3′; reverse 5′-GTA ATC TTG AAC GCG GAT ATG -3′), *DWARF4* (forward 5′-GTG CTG CCA TTC TCG GAG TAA TAG-3′; reverse 5′-CTC AGC AAG AGG TCC AGG ATT TGC-3′), *CPD1* (forward 5′-CGA CGG CCT TCT TCT CCA T-3′; reverse 5′-AGG GCC TGG CCG TAG GT-3′), *CPD2* (forward 5′-CAC CAC ACG AAC TCT CAA AGG A-3′; reverse 5′-GCA AAA CAA TCT AAC GTC AGC AA-3′), *DET2* (forward 5′-CCT CTG TTA TTG CTG ATG GAT ACG-3′; reverse 5′-ATG CCA ACA GTA TGA ATC AAA AGC-3′), *BRI1* (forward 5′-CAG CTT GGA GGA TGT GTT GCGGA-3′; reverse 5′-TCT TCG AGT CGA CCG TCG AC-3′), *XTR3* (forward 5′-ATC GGA GCA GCT AGC TAG AG-3′; reverse 5′-GTA GAA GGC AAC GAC GAC GC-3′), *BZR1* (forward 5′-ATG ACG GCC ATT ATT GCC GAG CA-3′; reverse 5′-TCG CCC AAA TCG CAG CAT-3′), *ACT1* (forward 5′-TGC TAT GTA CGT CGC CAT CCA G-3′; reverse 5′-AAT GAG TAA CCA CGC TCC GTC AA-3′).

### 3.8. Statistical Analysis

The data were analyzed using analysis of variance using IBM SPSS Statistics 23 software (IBM Corp. Armonk, NY, USA). Means with asterisks indicate significantly different values at *p* < 0.05 (*), or 0.001 (***) according to least significance difference (LSD) test. All data are presented as mean ± standard deviation.

## 4. Conclusions

Unlike melatonin production in animals, which peaks in the dark, melatonin biosynthesis in plants requires light. No melatonin was induced during dark incubation when rice plants were challenged with cadmium, whereas large amounts of melatonin were induced when plants were incubated in light [2]. From the results of more detailed experiments, we found that light quality played a role in light-induced melatonin biosynthesis, and the combination of red and blue light led to maximum melatonin production, followed by red light alone and blue light alone. In comparison, melatonin is not induced in rice with cadmium treatment upon exposure to far-red light and the dark. The role of light quality in melatonin synthesis was confirmed genetically, with rice phytochrome (red and far-red photoreceptors) mutants synthesizing less melatonin than the wild type, which implies that melatonin synthesis is light-dependent in plants [33]. Here, we tested whether the blue-light photoreceptor (CRY) is also involved in melatonin biosynthesis. We generated *CRY1b* RNAi rice and challenged rice plants with cadmium to induce melatonin. As expected, reduced melatonin synthesis was observed in the *CRY1b* RNAi lines compared to the wild type, providing further evidence for light-dependent melatonin biosynthesis in plants.

## Data Availability

The data presented in this study are available on request from the corresponding author.
